# The Phase Behavior of γ-Oryzanol and β-Sitosterol in Edible Oil

**DOI:** 10.1007/s11746-015-2731-3

**Published:** 2015-11-02

**Authors:** Hassan Sawalha, Paul Venema, Arjen Bot, Eckhard Flöter, Ruud den Adel, Erik van der Linden

**Affiliations:** Laboratory of Physics and Physical Chemistry of Foods, Department of Agrotechnology and Food Sciences, Wageningen University, Bornse Weilanden 9, 6708 WG Wageningen, The Netherlands; Unilever Research and Development Vlaardingen, Olivier Van Noortlaan 120, 3133 AT Vlaardingen, The Netherlands; Chemical Engineering Department, An-Najah National University, P.O. Box 7, Nablus, Palestine; Environmental Technology Engineering Department, Palestine Polytechnic University, Hebron, Palestine; Food Process Engineering, Department of Food Technology and Food Chemistry, Technical University Berlin, Königin-Luise-Strasse 22, 14195 Berlin, Germany

**Keywords:** Phase behavior, Phase diagram, Solidification, γ-Oryzanol, β-Sitosterol, Organogel, Oleogel, DSC, SAXS, Eutectic point, Tubule

## Abstract

The phase behavior of binary mixtures of γ-oryzanol and β-sitosterol and ternary mixtures of γ-oryzanol and β-sitosterol in sunflower oil was studied. Binary mixtures of γ-oryzanol and β-sitosterol show double-eutectic behavior. Complex phase behavior with two intermediate mixed solid phases was derived from differential scanning calorimetry (DSC) and small-angle X-ray scattering (SAXS) data, in which a compound that consists of γ-oryzanol and β-sitosterol molecules at a specific ratio can be formed. SAXS shows that the organization of γ-oryzanol and β-sitosterol in the mixed phases is different from the structure of tubules in ternary systems. Ternary mixtures including sunflower oil do not show a sudden structural transition from the compound to a tubule, but a gradual transition occurs as γ-oryzanol and β-sitosterol are diluted in edible oil. The same behavior is observed when melting binary mixtures of γ-oryzanol and β-sitosterol at higher temperatures. This indicates the feasibility of having an organogelling agent in dynamic exchange between solid and liquid phase, which is an essential feature of triglyceride networks.

## Introduction

Mixtures of plant sterols (e.g., γ-oryzanol and β-sitosterol) can be potentially used as an alternative to crystalline fats like triacylglycerols (TAGs) for structuring the oil phase of some food products. Certain crystalline TAGs contribute to raising blood cholesterol as a result of their high saturated fatty acids content and have been identified as a risk factor for cardiovascular diseases [1–4]. In contrast, reports showed that the intake of plant sterols has a blood cholesterol-lowering effect; for instance, the blood cholesterol content can be reduced by up to about 10 % upon a total intake of 2 g plant sterols/day [5]. Therefore, plant sterol-containing food products are expected to have a positive impact on human health. Recently, some studies reported on the use of plant sterol (ester + sterol) mixtures (γ-oryzanol and β-sitosterol) for the preparation of organogels and emulsions [6–12]. It was found that γ-oryzanol and β-sitosterol self-assemble into hollow double-walled tubules (ca. 10 nm in diameter) forming a transparent and firm organogel [10]. The formation of tubules is driven by enthalpy changes, indicating that the molecular interaction between γ-oryzanol and β-sitosterol determines the properties of the resulting gel to a large extent [12, 13]. The physical and mechanical properties of the gel, including firmness, transparency, melting and tubular microstructure, were found to be dependent on the γ-oryzanol to β-sitosterol ratio. For instance, the firmest gel was obtained with 1:1 molar ratio of γ-oryzanol to β-sitosterol and the gels rich in β-sitosterol were more hazy and had a higher melting point.

**Fig. 1:**
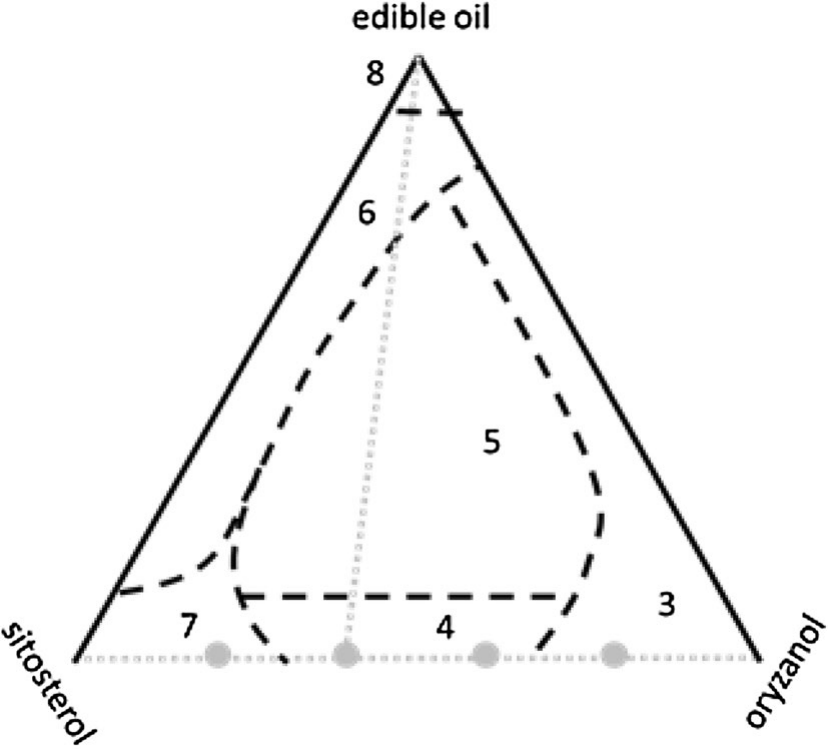
*Dashed black lines*: schematic representation of the ternary phase diagram of the β-sitosterol + γ-oryzanol + canola oil mixture according to AlHasawi and Rogers [14]. The diagram is based on weight percentages. The* numbers* in the figure indicate the regions belonging to different phases, and refer to the figure numbers in Ref. [14]. *Dotted gray lines*: ternary and binary mixtures as used in the present study, where sunflower oil was used as the edible oil. *Solid gray dots*: binary mixtures as used in melting experiments in the present study

Recently, AlHasawi and Rogers reported a qualitative study of the ternary phase diagram of β-sitosterol, γ-oryzanol, and edible vegetable oil [14]. Their results indicated that the phase behavior in these systems is quite rich (see Fig. 1), showing a relative large composition range compatible with the formation of organogels in the center of the phase diagram, but also a number of different areas with alternative phase behavior closer to the edges of the phase diagram. The area close to the pure β-sitosterol + γ-oryzanol axis of the diagram is of particular interest because it covers an expected transition from the oil-filled hollow tube to an alternative structure, since a hollow tubule is not likely to be stable in the absence of liquid oil. In fact, circumstantial evidence for an alternative structure can be found from the flattened structure of organogel tubules in de-oiled organogel samples [15].

The objective of the present paper was to study certain aspects of the ternary γ-oryzanol + β-sitosterol + edible oil phase diagram in more detail. Two specific questions were addressed: (1) How do the properties of binary γ-oryzanol and β-sitosterol mixtures change as a function of the ratio of both components? In particular, the question of the whether the microstructure of the mixture changes gradually as a function of composition or whether sudden transitions occur is of interest. (2) How does the structure of the tubules in the ternary mixture of ternary γ-oryzanol + β-sitosterol + oil change if the amount of liquid oil is gradually reduced? The boundaries are schematically indicated by the dotted lines in Fig. 1. These questions were addressed using differential scanning calorimetry (DSC) and small-angle X-ray scattering (SAXS).

## Experimental

### Materials

The γ-oryzanol (Tsuno Rice Fine Chemicals, Wakayama, Japan) and tall oil sterol (78.5 % β-sitosterol, 10.3 % β-sitostanol, 8.7 % campesterol, and 2.5 % of other minor sterols, Unilever, the Netherlands) were used in this study to prepare the binary mixtures. The organogels were prepared with sunflower oil (Reddy, NV Vandemoortele, Breda, the Netherlands) as a lipid phase. All materials were used as received.

## Methods

### Preparation of Binary γ-Oryzanol + β-Sitosterol Mixtures

To prepare well-mixed binary mixtures, the γ-oryzanol powders and β-sitosterol granules were first melted at ca. 165 °C in the oven and the molten solution was manually mixed. The mixed solution was then left overnight in the oven (the oven was switched off) to solidify while cooling to room temperature. The solidified binary mixture was manually ground and was ready for characterization with DSC and SAXS. The pure compounds were also subjected to the same treatment as of the binary mixtures.

### Preparation of Organogel

For the preparation of the organogel, γ-oryzanol and β-sitosterol were dissolved in sunflower oil using a magnetic stirrer equipped with a heater. The hot solution was subsequently cooled down until a gel was formed and stored in the fridge at 4 °C for at least 1 week before characterization. Organogels with different γ-oryzanol to β-sitosterol ratios and different total sterol concentrations were prepared.

### Differential Scanning Calorimetry (DSC)

Thermal transitions in binary γ-oryzanol/β-sitosterol mixtures and organogels were studied using a differential scanning calorimeter (Perkin Elmer Diamond DSC, Perkin-Elmer Co., Norwalk, CT). The ground binary mixtures or organogels samples (7–15 mg of each sample) were sealed in stainless steel cups and then scanned with the DSC system. Binary mixtures were scanned using the following procedure: heating from 5 to 160 °C, hold at 160 °C for 10 min, then cooling from 160 to 0 °C, hold at 0 °C for 60 min, and finally heating again from 5 to 160 °C. All heating/cooling rates were 10 °C/min. For some of the binary mixtures, an annealing step was applied in which the sample was cooled down from 160 to 90 °C, then kept at 90 °C for 300 min after which it was further cooled down to 0 °C. Previous work had shown that a temperature-holding step reduced the risk of entering a metastable state in oryzanol-rich samples.

The organogels were scanned with a slightly different program: heating from 0 to 120 °C, hold at 120 °C for 10 min, then cooling from 120 to 0 °C, hold at 0 °C for 10 min, and reheating from 10 to 120 °C with 10 °C/min as the heating/cooling rate.

### X-ray Scattering

Small-angle and wide-angle X-ray scattering (SAXS, WAXS) experiments were performed at the high-brilliance ID2 beamline of the European Synchrotron Radiation Facility (ESRF) in Grenoble, France [16]. Details of the experimental setup are given elsewhere [8]. SAXS/WAXS data were collected in two runs in either the range 0.079 nm^−1^ < *q* < 4.7 nm^−1^ and 3.1 nm^−1^ <* q* < 29.5 nm^−1^ or 0.094 nm^−1^ <* q* < 4.5 nm^−1^ and 3.9 nm^−1^ <* q* < 42.2 nm^−1^, respectively, where *q* is the scattering vector defined by *q* = 4*π*·sin*θ*/*λ* (with *θ* the scattering angle and *λ* the wavelength of the incoming X-ray beam). Scattering data were corrected for scattering from the oil phase by subtraction of the pure oil signal.

A number of complementary X-ray diffraction (XRD) measurements were performed (at Unilever R&D Vlaardingen) using a Bruker D8-Discover in a *θ*/*θ* configuration. Data was collected in the range 0.64 nm^−1^ < *q* < 7.7 nm^−1^ [11]. Data in these complementary experiments were not corrected for the contribution of oil.

## Results and Discussion

### Binary Mixtures

**Fig. 2:**
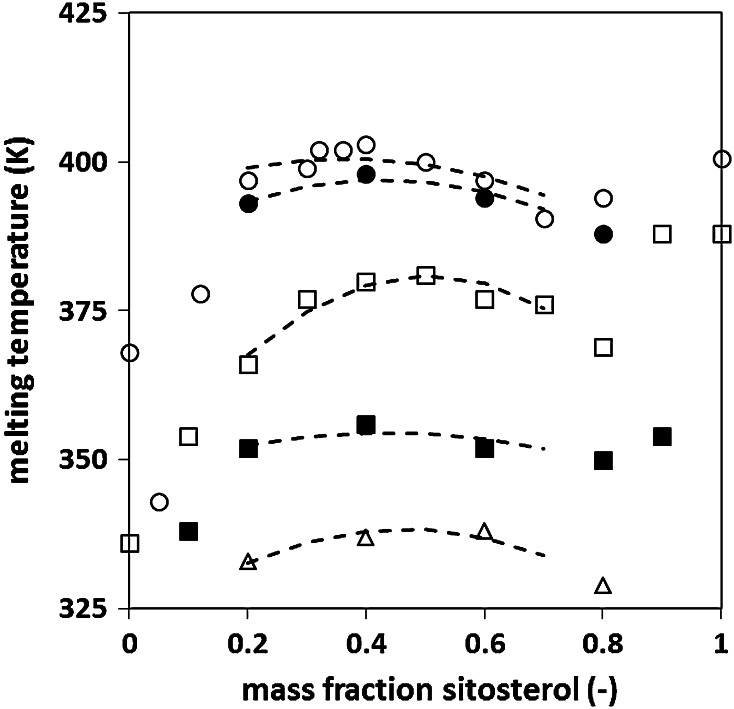
Melting temperature of γ-oryzanol + β-sitosterol binary mixtures and ternary mixtures in sunflower (organogel) as a function of β-sitosterol weight fraction in the sterol mixture and oil concentration. The data shown in this figure were obtained from the second heating DSC scans. Oil in sample: *circles* 0 %, *filled circles* 10 %, *square*s 60 %, *filled squares* 84 %, *triangles* 92 %. The *dashed lines* emphasize the presence of a local maximum in the melting curves. Together with the lowering of the melting point of the pure components this leads to a W-shaped melting curve

Binary γ-oryzanol + β-sitosterol mixtures were studied as a function of the ratio of the two components to determine their mixing behavior. At least two “minimal” scenarios can be envisioned (see Fig. 2: first curve from the top). The maximum in the melting temperature in the intermediate composition range is obviously related to congruent melting. However, the solid phase melting at this point can either be a compound, a mixed crystal with a defined stoichiometric γ-oryzanol to β-sitosterol ratio, or analogous to an azeotrope an energetically favorable mixed crystal that occurs within a specific compositional range. The last of these is also referred to as hylotrope. Since γ-oryzanol and β-sitosterol in ternary systems with oil form specific tubular assemblies at a 1:1 molar ratio in the central region of the phase diagram (see Fig. 1), the formation of a stoichiometric compound appears in the first place more likely. In essence a compound splits the phase diagram of binary mixtures into two binary systems, namely compound plus component A and compound plus component B. This implies that for system compositions neighboring the stoichiometric ratio of the compound two two-phase regions have to exist. Consequently these neighboring compositions will crystallize as two distinct solid structures at proportions dictated by simple mass balances. In contrast to this mixed crystals of γ-oryzanol + β-sitosterol formed over a composition range will show a gradual change in structure as a function of compositional changes [17].

The underlying mechanism can be studied by a combination of both DSC and SAXS [18].

### Thermal Behavior of Pure Compounds and Binary Mixtures (DSC Data)

**Fig. 3:**
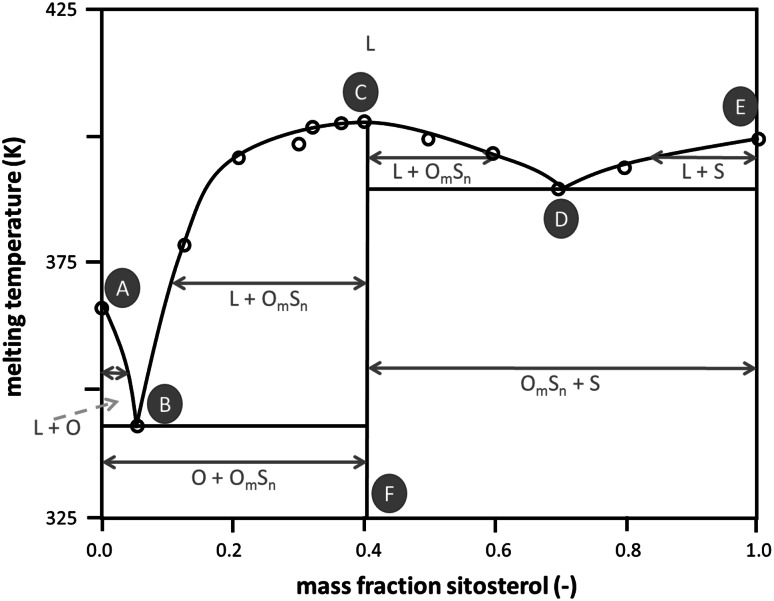
Simplified binary phase diagram for the binary γ-oryzanol + β-sitosterol system, as inferred from the DSC data. The* vertical line CF* indicates the existence of a compound, the* curve through points ABCDE* is the liquidus, the area below the liquidus consists of two-phase areas of the compound O_*m*_S_*n*_ and either sitosterol (S) or oryzanol (O). For details, see text

The melting temperatures for binary γ-oryzanol + β-sitosterol mixtures are shown in Fig. 3. It can be seen that the curve has an overall W shape, with a maximum at approximately 40 % and two minima at 5 and 70 % (w/w) β-sitosterol. It should be noted that both pure components but especially the γ-oryzanol-rich compositions have a tendency to remain in metastable states on the time scale of a DSC experiment, leading to an initial underestimation of the temperature of melting for those compositions. Repeated annealing of the mixtures or pure components changes the temperature towards the melting temperature of the most stable polymorphic form. This, however, complicates the determination of the phase behavior of the binary mixtures greatly. The W shape suggests that both subsystems, compound plus γ-oryzanol and compound plus β-sitosterol, show eutectic behavior. This implies that around the composition of the temperature minima, 5 and 70 % (w/w) β-sitosterol, two solid–solid two-phase regions exist.

On the basis of the DSC data and assuming the formation of a 1:1 molar compound (40 % w/w β-sitosterol), the binary phase diagram as shown in Fig. 3 can be constructed. Depending on composition and temperature the phase diagram has the following properties: a completely mixed liquid phase containing components O (γ-oryzanol) and S (β-sitosterol) above the liquidus (curve ABCDE), four liquid–solid two-phase regions, and two solid–solid two-phase regions. Point A indicates the melting point of pure component O, while point E indicates the melting point of pure component S. The temperature maximum also denoted as a dystectic point is indicated by point C. As suggested by the experimental data and dictated by theory (e.g., Nerad *et al*., 18), a smooth curve is drawn around the dystectic point. As pointed out above the compound separates the binary system into two “pseudo-binary” subsystems. Similar to many phase diagrams revealed in lipid science there is no information on the state of mixing in the solid phases and the diagram here hence assumes complete immiscibility. This means that at temperatures lower than given by the eutectic point B the solids in the mixed system are dependent on the composition, i.e., either the solid compound coexisting with pure solid γ-oryzanol or solid compound coexisting with pure solid β-sitosterol.

### SAXS Data of Binary Mixtures

**Fig. 4:**
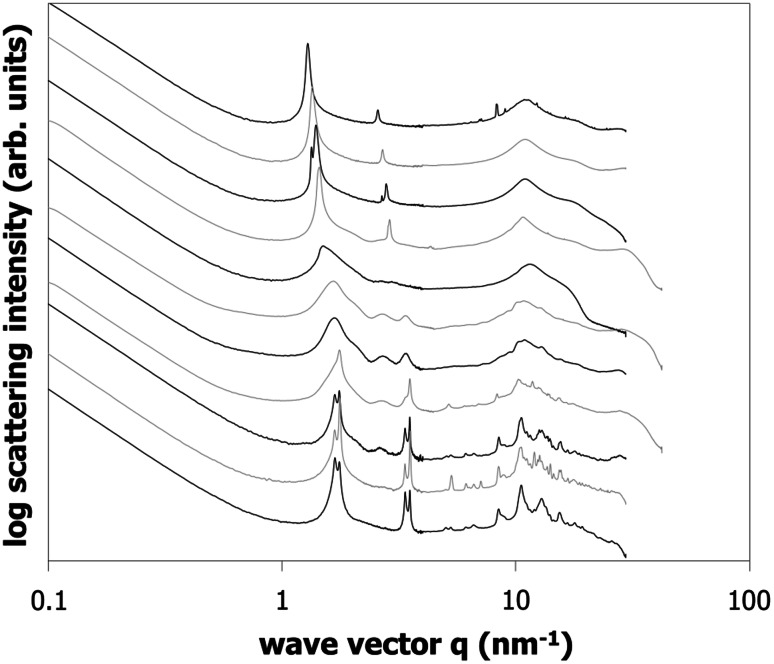
SAXS data of different γ-oryzanol + β-sitosterol binary mixtures. Data taken at 10 °C after at least 1 week storage at 5 °C. γ-Oryzanol to β-sitosterol mass ratio from* top* to* bottom*: 100:0, 90:10, 80:20, 70:30, 60:40, 50:50; 40:60, 30:70, 20:80, 10:90, and 0:100

SAXS is a suitable tool to elucidate the nature of the composition-dependent structures in the binary mixture of γ-oryzanol + β-sitosterol. In principle, the SAXS data of powdered γ-oryzanol and β-sitosterol after long storage are known from the literature [19]. For the annealed samples in the present study, the scattering pattern for β-sitosterol agrees with the one determined previously for the powdered sample (see Fig. 4). The scattering pattern for the γ-oryzanol differs, however, and agrees more with the pattern observed for the system (in an emulsion) after recrystallization. This reconfirms that the present system tends to remain in metastable states for the mixtures rich in γ-oryzanol.

For the γ-oryzanol + β-sitosterol mixtures, Fig. 4 shows three regions. First of all, a region for compositions rich in β-sitosterol can be identified (ca. 70–100 % β-sitosterol), in which sharp peaks associated with the pure β-sitosterol crystal structure can be observed.

A similar region can be found for ca. 70–100 % γ-oryzanol, although the scattering shows fewer sharp features than for β-sitosterol—especially in the WAXS range around 10 nm^−1^. It is interesting to note that the main scattering peak at ca. 1.4 nm^−1^ shifts to slightly higher *q* values from 1.29 to 1.44 nm^−1^ when the γ-oryzanol is diluted in β-sitosterol. Using *d* = 2*π*/*q*_*i*_, where *q*_*i*_ identifies the wave vector peak position associated with a particular peak, this indicates that the average bilayer distance decreases from 4.89 to 4.35 nm as would be expected as a result of the mixing in of a shorter molecule (a weak bilayer peak in γ-oryzanol powder can be found at 4.95 nm^−1^, whereas for β-sitosterol a bilayer spacing of ca. 3.8 nm^−1^ is expected [19]). The second, weaker peak at ca. 2.7 nm^−1^ originates from half the bilayer distance.

The third region in Fig. 4 shows qualitatively different behavior from the pure substances. Although we have not been able to quantitatively interpret the scattering data, it is clear that the pattern is much closer to that observed for the tubules in the organogel. One hypothesis is that the data reflects a flattened version of the tubules, as no liquid oil is present to fill the tubules. Tentative support for this interpretation can be obtained from SEM images of somewhat flattened tubules in de-oiled samples [15].

As pointed out above, the phase behavior as shown in Fig. 3 is solely based on DSC data. Combining the SAXS data with some general knowledge it is fairly straightforward to further detail the phase behavior. The assumption of complete immiscibility has to result in a very regular evolution of the scattering data. In this case the scattering data should be a simple linear combination of the pattern of the two solid structures coexisting which each other. In contrast it turns out on combination of diffractograms that none of the intermediate scattering patterns relating to compositions between 0 to 40 % (w/w) β-sitosterol can be made up from the patterns of the compound crystal and the pure γ-oryzanol. As mentioned before, the data rather suggest a gradual change as a function of the β-sitosterol inclusion level. However, the existence of a single mixed crystalline structure over the range from 0 to 30 % w/w) β-sitosterol inclusion is in conflict with the rules of equilibrium phase behavior. Starting from point B (eutectic) lower temperatures have to relate to a region of two coexisting solid phases. This conflict is possibly resolved by accepting that the DSC and X-ray data of pure γ-oryzanol relate to different crystalline structures. It appears that the possibly stable structure found in the mixing range from more than 5 % to more than 30 % (w/w) β-sitosterol extends metastably into the range of higher γ-oryzanol concentrations.

**Fig. 5:**
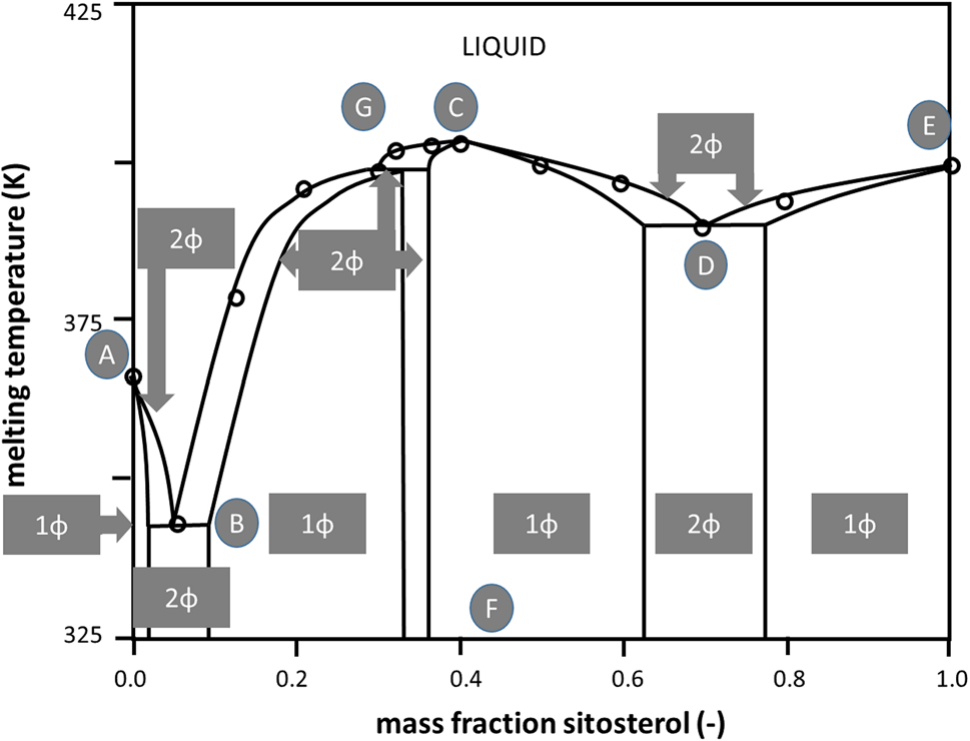
Full binary phase diagram for the binary γ-oryzanol + β-sitosterol system based on both DSC and X-ray scattering data.* Number* *ϕ* indicates the number of coexisting phases. The one-phase (1*ϕ*) compositions have broadened to regions of finite width compared to the simplified diagram, the* curve through points ABGCDE* is the liquidus, the two-phase (2*ϕ*) areas below the liquidus have shrunk. For details, see text

For concentrations rich in β-sitosterol it appears that up to 20 % (w/w) of γ-oryzanol can be hosted by the crystalline structure. The data analysis further confirms that there is a solid–solid two phase region expanding around the composition of the eutectic point at 70 % (w/w) β-sitosterol (point D, Fig. 3). This is so because the scattering pattern of this sample can almost perfectly be matched by a 1:1 linear combination of the scattering patterns of the samples with 60 and 80 % (w/w) β-sitosterol, respectively. In the intermediate composition range of 40 to 60 % (w/w) of β-sitosterol, another distinct scattering pattern exists. Within this composition range the structure appears to change gradually, indicating a one-phase region with a mixed crystalline phase. This suggests that the temperature maximum in Fig. 3 (point C) is rather related to a hylotrope than to a stoichiometric compound. Inclusion of this interpretation into a more comprehensive phase diagram makes it is necessary to introduce another two-phase region that separates the two intermediate one-phase regions, one being γ-oryzanol rich and the other located around the equimolar composition (point C, Fig. 3). The suggested significantly more complex phase behavior is depicted in Fig. 5. Actually the presence of point G in Fig. 3, not perfectly matching the liquidus line, supports this change to the phase behavior proposed. The existence of a peritectic point introduced matches with the discontinuity of the liquidus line due to incongruent melting.

The identified positions of the transitions are in qualitative agreement with the data by AlHasawi and Rogers [14]. These authors indicate transitions at approximately 30 and 70 % β-sitosterol. In the range of high γ-oryzanol concentrations these authors did not identify a transition. However, as pointed out above the system is prone to remain in metastable states. Consequently is the transition at high γ-oryzanol concentrations (around 95 %, w/w) primarily a must dictated by the clearly identified eutectic point.

However, the complexity introduced on the basis of the SAXS data and displayed in Fig. 5 is in better agreement with the work of AlHasawi and Rogers [14] than the phase diagram shown in Fig. 3.

### Ternary Mixtures (Organogels)

The studies on the binary mixtures established that oil-free mixtures below the melting temperature do not show the features of full tubule formation despite some features in the scattering data being reminiscent of the sterol tubules. The next stage is to proceed to mixtures containing edible oils to study the transition from binary mixture to tubules in the presence of liquid oil.

### Thermal Behavior of Ternary Mixtures (Organogel)

**Fig. 6:**
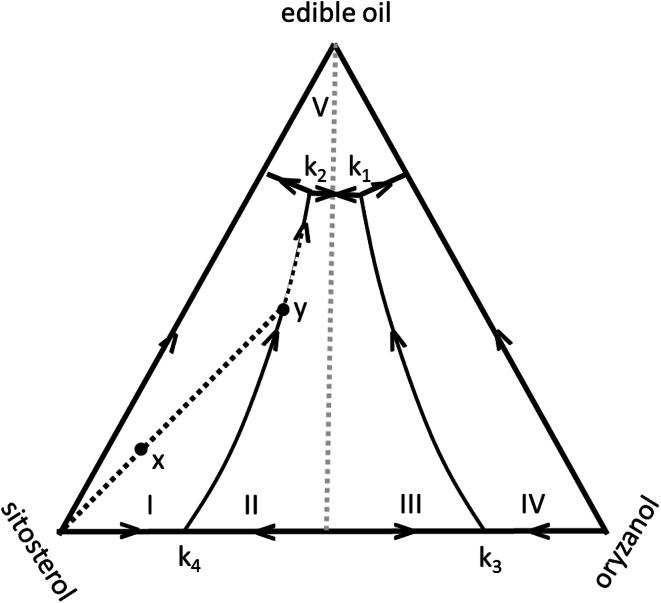
Schematic projection of the melting plane for the γ-oryzanol + β-sitosterol + sunflower oil ternary mixture assuming a binary phase behavior as described in Fig. 3. The *dashed line* *xyk*_*2*_ describes the solidification trajectory of a sample of composition *x* in the melting plane,* lines* *k*_*1*_ *k*_*2*_ and* k*_*3*_ *k*_*4*_ connect the binary eutectic points in the diagram, the *dotted vertical line* represents the composition of the compound (the *dotted line* would be drawn at an angle as in Fig. 1 if the intention had been to draw a semiquantitative diagram)

Figure 2 shows the melting curves of the ternary mixtures. The addition of oil depresses the melting point of the binary mixture (the first four curves from the bottom), but the overall shape of the melting curves remains similar. This observation allows us to sketch the ternary phase behavior, at least in a semiquantitative way [20]. Figure 6 shows the projection of the melting plane from the temperature–composition phase on the composition triangle. As a result each point in this triangle corresponds to the specific melting temperature belonging to the mixture in that point. The arrows on the phase boundaries indicate in which direction the composition of the liquid mixture changes upon cooling. The O–S (γ-oryzanol to β-sitosterol) axis corresponds to the melting curve of the binary mixture (see Fig. 3 or 4). This type of diagram is especially useful to follow the solidification (or melting) path of a specific mixture. Since assuming either a phase behavior according to Fig. 3 or 5 would only marginally change Fig. 6, the discussion of Fig. 6 will be based exclusively on Fig. 3 for reasons of simplicity. For example, when a sample with composition x is sufficiently cooled, it will eventually reach its crystallisation point (point x in Fig. 6) and S crystals will be formed. Upon further cooling, the composition of the liquid gets richer in edible oil and O and the composition of the liquid mixture moves towards point y located on the phase boundary k_4_–k_2_. Further cooling after reaching point y, both S crystals and the solid compound O_*m*_S_*n*_ will be formed, making the composition of the liquid richer in edible oil. Upon cooling the composition will move along the line k_4_–k_2_, until point k_2_ is reached. This is the ternary eutectic point where all components will be solidified provided the sample is sufficiently cooled to allow for the eutectic reaction to take place.

The diagram in Fig. 6 predicts a central region dominated by the O_*m*_S_*n*_ compound, in combination with either S or O crystals. Along the axes of the diagram we find O crystals along the O–Oil line, and S crystals along the S–Oil line. The line along the O–S axis passes for the most part through a range for the O_*m*_S_*n*_ compound, except at the edges which are again dominated by pure crystals (either O or S). For the oil-free cases, region I corresponds to line AB, region II to line BC, region III to line CD, and region IV to line DE in Fig. 3, but extends also along the oil axis. Close to the oil vertex, there is a small region which reflects the crystallization of the edible oil at low* T*. Since the ternary mixtures are not cooled below 0 °C, the oil remains liquid and the top part of the diagram in Fig. 6 remains unexplored. We can compare Fig. 6 to the ternary phase diagram as presented by AlHasawi and Roger [14] (Fig. 1 as obtained by different experimental techniques). It should be realized that the microscopic images in their diagram are of the final structure at 30 °C, while the diagram in Fig. 6 allows for following the crystallization path upon cooling.

### SAXS Data of Ternary Mixtures (Organogel)

**Fig. 7:**
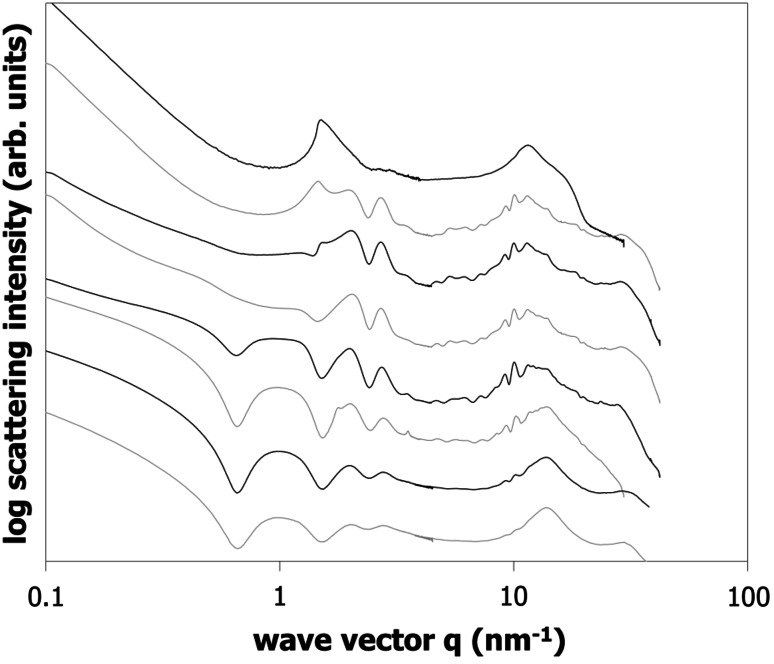
SAXS data of organogels prepared with (60:40 w/w mixture of γ-oryzanol/β-sitosterol) in sunflower as a function of oil concentration. Data taken at 10 °C after at least 1 week storage at 5 °C. From* top* to* bottom*: 0, 5, 10, 20, 40, 68, 84, and 92 % oil (w/w)

Figure 7 shows the scattering pattern for a dilution series of 60:40 % γ-oryzanol/β-sitosterol mixtures in sunflower oil. The data shows the intermediate stages between the oil-free sample shown in Fig. 4 and the interference patterns due to tubules from earlier publications [8, 9, 15]. The oil-free sample shows a feature at *d* = 2*π*/*q*_*i*_ = 4.19 nm. This feature has not been observed previously in β-sitosterol, γ-oryzanol, or their mixture in oil, water, or emulsions [19].

A pattern builds up from 5 % towards 40 % oil, starting from the small* d* peak but involving more and more larger* d* peaks at increasing oil content (in order of appearance: *d* = 2.30 nm, *d* = 3.15 nm, and *d* = 6.53 nm) under simultaneous weakening of the 4.19-nm feature. Taking the *d* = 2.30 nm peak as the shortest distance, the sequence of ratios of the *d* values for 5 and 10 % oil (1:1.37:1.83) comes close to the 1:√2:√3 sequence of a liquid crystalline cubic structure, as was noted before by AlHasawi and Rogers [14]. Given the fact that the smallest two* d *values coincide with those observed later at higher dilutions for tubules, the tentative cubic structure probably shares some elements with the tubules. The commonality should occur at a length scale well below a single twist of a helical ribbon (i.e., no more than a few stacked molecules); however, model calculations show that even very short tubules show all the reflections of infinitely large tubules. We therefore do not expect that these tentative cubic structures can be assigned to the full central region in the phase diagram and also because there is ample supporting evidence that tubule formation occurs over most part of the central region of the phase diagram [15]. For 20 % oil, the appearance of a peak close to *d* = 6.5 nm completes the set of features that characterize the tubule diffraction, albeit at different intensities than in a fully developed interference pattern due to tubules. The simultaneous intensity increase of the peaks associated with tubules with an intensity decrease of the *d* = 4.19 nm feature suggests the coexistence of two different structures in these systems with small amounts of oil.

It is possible to provide a rough estimate of the amount of oil needed to develop the tubules fully. Assuming the parameters obtained from earlier neutron scattering experiments gives *r*_in_ = 2.43 nm and *r*_out_ = 4.74 nm [10]. Assuming maximum hexagonal packing of perfectly aligned tubules and using (1 − *π*√3/6) + (*π*√3/6) · (*r*_in_/*r*_out_)^2^ = 0.33, it is found that a minimum oil content of about 33 % is needed to develop the tubules fully (where we ignore the fact that the outside tubule has a lower density because only half of the molecules in the β-sitosterol + γ-oryzanol tubule contain the ferulic acid moiety).

Indeed, Fig. 7 shows that the tubule scattering pattern is found at oil contents of 40 % and above, which is consistent with this lower boundary. Note that the apparent loss in intensity of the peaks associated with the *d* = 2.30 nm and 3.15 nm features is due to an increase in intensity of a broad liquid oil feature, which has not been subtracted.

The slope at low* q* indicates that formation of linear structures (like tubules) occurs from 10 % oil onwards. In addition, it is noteworthy—though mentioned previously [8]—that the WAXS area lacks any sharp features for the samples with 92 % oil, indicating the absence of long-range translational crystalline order in these systems.

### Melting Behavior of Binary Mixtures

The next interesting question that is raised by these results is whether tubule formation requires liquid triglyceride oil. In a way, this question has been answered already in a previous study, in which it was shown that tubules are formed in a range of apolar fluids [15]. These results suggest that tubule formation would also occur in a fluid γ-oryzanol + β-sitosterol mixture. This idea can be tested by heating a binary γ-oryzanol + β-sitosterol solid mixture and seeing whether tubule formation occurs spontaneously when sufficient γ-oryzanol and/or β-sitosterol is in the liquid state creating mobility for the molecules.

**Fig. 8:**
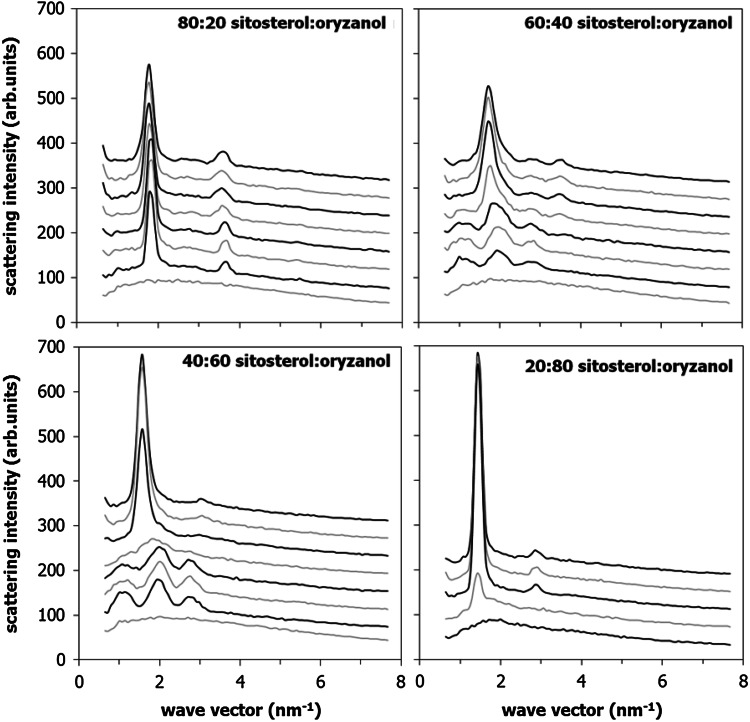
SAXS data of melting binary 20:80, 40:60, 60:40, and 80:20 γ-oryzanol + β-sitosterol mixtures in the temperature range from* top* to* bottom* between 20, 40, 60, 78, 95, 112, 127, and 144 °C (for the* bottom right figure* the three highest temperatures are left out because they are above the melting temperature). Curves have been shifted vertically for convenience

The data in Fig. 8 show SAXS scattering patterns for 20:80, 40:60, 60:40, and 80:20 γ-oryzanol + β-sitosterol binary mixtures. The data were obtained on a lower resolution SAXS setup because the long heating and cooling cycles make inefficient use of the limited measurement slots at the synchrotron. The 20:80 and 80:20 mixtures show patterns that are clearly reminiscent of the patterns associated with the pure components, β-sitosterol and γ-oryzanol, respectively. Upon heating, the pattern does not change quantitatively, although the features decrease in intensity as the mixed solids melt on temperature increase. The behavior of the 40:60 and 60:40 mixtures is more interesting, and was expected to be qualitatively different on the basis of the phase diagram in Fig. 1. At lower temperatures, the pattern is still relatively close to the pattern of the pure components, although some features observed in Fig. 4 (line broadening and shifting) appear here as well. This shift in the refraction pattern between different mixtures again supports the phase behavior as proposed in Fig. 5. Upon heating, however, a completely new pattern emerges at 80–100 °C. The pattern appears to be similar to that of equimolar γ-oryzanol + β-sitosterol mixtures in 10–40 % oil.

Analysis of the DSC curves for these samples during the first heating suggest that 2–15 % of the β-sitosterol + γ-oryzanol mixture is liquid over the temperature range between 80 and 100 °C. This figure is, however, quite sensitive to the choice of the baseline. The discussed occurrence of a liquid phase is actually in conflict with the phase diagram depicted in either Fig. 3 or 5 because at temperatures below the solid–liquid two-phase regions no liquid should be present. A meaningful explanation for the presence of a “low-temperature” liquid phase is based on the assumption that the phase behavior is actually more complicated than depicted in Fig. 3 or 5. The occurrence of this liquid could be the result of melting of a metastable polymorphic form combined with the slow crystallization of the more stable polymorph that has been the subject of the DSC analysis that Figs. 3 and 5 are based on.

Although the transition ranges for the dilution and melting experiments do no match exactly, the qualitative agreement between the ternary system and the system in melt transition is sufficient to argue that the liquid mixtures of β-sitosterol and γ-oryzanol function similarly to liquid triglyceride oils in promoting tubule formation.

## Conclusions

The present paper investigated the phase behavior of binary and ternary mixtures of γ-oryzanol + β-sitosterol with and without sunflower oil. It complements the earlier study by AlHasawi and Rogers [14], which investigated a larger area of the phase diagram, in the sense that the present study takes a deeper look into a few specific changes in these systems and does not consider the full phase diagram of the ternary system. Overall, both studies are in agreement.

The melting behavior of the binary mixtures indicates the presence of two eutectic points and shows a maximum dissolution temperature. A phase diagram describing the melting points was derived on the basis of the DSC data. However, taking into account the DSC data together with the SAXS data affords a more complex phase diagram that is in agreement with the data presented by AlHasawi and Rogers [14] and basic thermodynamical phase rules. The suggested phase diagram contains four distinct mixed solid phases that are separated by three two-phase regions located around 5, 30, and 70 % (w/w) β-sitosterol. Next to the two eutectic points identified earlier the complex phase diagram suggests the presence of a peritectic point. It has to be acknowledged though that the SAXS data and melting points (DSC) are gathered at significantly different temperatures. Furthermore SAXS shows that the organization of γ-oryzanol and β-sitosterol in the intermediate composition range is different from the structure of the tubules found in oil-diluted samples.

The phase behavior of the ternary mixtures does not indicate a sudden transition from binary compound to tubule. Rather, a gradual transition seems to occur as more and more liquid oil becomes available on dilution of the γ-oryzanol + β-sitosterol mixtures with edible oil.

Finally, it seems that this behavior can also be observed in binary mixtures of γ-oryzanol and β-sitosterol. Once a mixed liquid phase is present, as for example on the disintegration of mixed metastable polymorph, the same behavior as in oil-diluted systems is found. This finding is important because it suggests that in the organogel systems the sterol (ester) molecules are dynamically transferring from tubules to solution and *vice versa*.
